# Human adult mesangiogenic progenitor cells reveal an early angiogenic potential, which is lost after mesengenic differentiation

**DOI:** 10.1186/s13287-017-0562-x

**Published:** 2017-05-02

**Authors:** Marina Montali, Francesca M. Panvini, Serena Barachini, Francesca Ronca, Vittoria Carnicelli, Stefano Mazzoni, Iacopo Petrini, Simone Pacini

**Affiliations:** 1 Department of Clinical and Experimental Medicine, Hematology Division, University of Pisa, Via Roma 56, 56126 Pisa, Italy; 2 Department of Surgical, Medical and Molecular Pathology and Critical Care Medicine, University of Pisa, Pisa, Italy; 3 Department of Translational Research and New Technology in Medicine, University of Pisa, Pisa, Italy

**Keywords:** Mesangiogenic progenitor cells, Angiogenesis, Mesenchymal stromal cells, Nestin, Bone marrow-derived cells, Sprouting, TEM1, DLL-4, Chorioallantoic membrane assay

## Abstract

**Background:**

Mesangiogenic progenitor cells (MPCs) have shown the ability to differentiate in-vitro toward mesenchymal stromal cells (MSCs) as well as angiogenic potential. MPCs have so far been described in detail as progenitors of the mesodermal lineage and appear to be of great significance in tissue regeneration and in hemopoietic niche regulation. On the contrary, information regarding the MPC angiogenic process is still incomplete and requires further clarification. In particular, genuine MPC angiogenic potential should be confirmed in-vivo.

**Methods:**

In the present article, markers and functions associated with angiogenic cells have been dissected. MPCs freshly isolated from human bone marrow have been induced to differentiate into exponentially growing MSCs (P2-MSCs). Cells have been characterized and angiogenesis-related gene expression was evaluated before and after mesengenic differentiation. Moreover, angiogenic potential has been tested by in-vitro and in-vivo functional assays.

**Results:**

MPCs showed a distinctive gene expression profile, acetylated-low density lipoprotein uptake, and transendothelial migration capacity. However, mature endothelial markers and functions of endothelial cells, including the ability to form new capillaries, were absent, thus suggesting MPCs to be very immature endothelial progenitors. MPCs showed marked 3D spheroid sprouting activating the related molecular machinery, a clear in-vitro indication of early angiogenesis. Indeed, MPCs applied to chicken chorioallantoic membrane induced and participated in neovessel formation. All of these features were lost in mesengenic terminally differentiated P2-MSCs, showing definite separation of the two differentiation lineages.

**Conclusion:**

Our results confirm the bona-fide angiogenic potential of MPCs and suggest that the high variability reported for MSC cultures, responsible for the controversies regarding MSC angiogenic potential, could be correlated to variable percentages of co-isolated MPCs in the different culture conditions so far used.

**Electronic supplementary material:**

The online version of this article (doi:10.1186/s13287-017-0562-x) contains supplementary material, which is available to authorized users.

## Background

In a research study aimed to isolate human bone marrow-derived mesenchymal stromal cells (MSCs) for clinical applications we identified a novel cell population, specifically isolated in pooled human AB serum (PhABS) supplemented medium. Initially, we named these cells mesodermal progenitor cells (MPCs) for their ability to retain mesengenic, angiogenic, and apparently cardiomyogenic potential after multiple steps of differentiation [1]. Later, a revised nomenclature for these cells was proposed due to the lack of convincing data about genuine differentiation toward cardiomyocytes. Thus, these cells were renamed mesangiogenic progenitor cells, maintaining the acronym MPCs [2]. MPCs showed morphological, phenotypic, and molecular features different from MSCs. In particular, they were characterized by their distinctive gene expression profile [3] and growing/adhesion properties [4].

In order to enrich bone marrow mononuclear cell (BM-MNC) cultures in MPCs we established selective conditions that allowed us to obtain almost pure MPC cultures [2, 5]. MPC differentiation toward MSC-like cells was investigated and we were able to finely dissect the pathway, including definition of single steps and timing [6].

On the other hand, MPC induction toward the endothelial lineage still retains some open issues. The protocol we set up to test MPC angiogenic potential included the “pre-differentiation” EndoCult® medium (StemCell, Vancouver, Canada), designed to support colony forming unit-Hill (CFU-Hill) cells but unsuitable to culture mature endothelial cells [7]. MPC-derived predifferentiated cells were CD90 positive, CD31 negative, and KDR/Flk-1 partially positive [5]. The formation of capillary-like structures (CLS) in Matrigel® was identified but we were unable to show unambiguously that CLS originated from terminal differentiation of CD90^+^CD31^neg^KDR^+^ cells. In fact, uncontrolled mesengenic differentiation in EndoCult® preconditioning medium allows CD90^+^CD31^neg^KDR^neg^ MSC-like cells to grow. They would be responsible for formation of CLS by differentiating directly into endothelial cells, as suggested by recent investigation [8–10].

In order to further clarify the issue and to detail the endothelial differentiation pathway, we analyzed the angiogenic potential of both highly purified MPCs and MPC-derived MSCs.

## Methods

### Primary cell cultures

#### Donors

Bone marrow samples were collected, after written consent, from 12 patients (four male/seven female, median age 63 years) during orthopedic surgery for hip replacement. A 20-ml syringe containing 500 U.I. of heparin was used to aspirate 10 ml of fresh tissue immediately after femoral neck osteotomy and before femoral reaming. Samples were processed soon after.

#### MPC cultures

Fresh bone marrow samples were diluted 1:4 in Dulbecco’s Modified Phosphate Buffer (D-PBS; LifeTechnologies, Carlsbad, CA, USA) and gently layered on Ficoll-Paque™ PREMIUM (GE Healthcare, Uppsala, Sweden). After centrifugation at 400 × *g* for 25 min, human BM-MNCs were harvested at the interface, filtered on 50-μm filters, and washed twice in D-PBS. Cells were seeded (8 × 10^5^ cells/cm^2^) on hydrophobic plastics in Dulbecco’s Modified Minimal Eagle’s Medium (DMEM) supplemented with 1% Glutamax®, 1% penicillin–streptomycin (LifeTechnologies), and 10% pooled human AB-type serum (PhABS) of US origin (Lonza, Walkersville, MD, USA). Nonadherent cells were removed after 48 h and cultures were maintained for 6 days with further passaging at day 4. MPCs were harvested by TryPLE Select® (LifeTechnologies) digestion.

#### Establishment of MSC cultures from MPCs

To induce differentiation into MSCs, freshly isolated MPCs were plated (2 × 10^4^ cells/cm^2^) in TC-treated T75 flasks and left to adhere overnight in 10% PhABS DMEM. The medium was then replaced by MesenPRO® Reduced Serum (RS) Medium (LifeTechnologies) and the cells grown to confluence (P1-MSCs), refreshing the medium every 2 days. P1-MSCs were detached by TryPLE Select® and subcultured to confluence (P2-MSCs).

#### Human umbilical vein endothelial cell culture

Human umbilical vein endothelial cells (HUVECs) were obtained, after written consent, as described previously [11] with slight modifications. Briefly, umbilical veins were perfused with 30 ml of 1% bovine serum albumin (BSA; Sigma-Aldrich, St. Louis, MO, USA) DMEM, filled with collagenase solution, and incubated for 30 min at 37 °C. Cell suspensions were then allowed to flow out by perfusion with an additional 30 ml of 1% BSA DMEM, washed twice, plated in T75 culture flasks coated with Attachment Factor (AF) Protein (LifeTechnologies), and passaged twice in vascular endothelial growth factor (VEGF)-rich endothelial growth medium (EGM-2; Lonza).

### Cell characterization

#### Flow cytometry

Freshly isolated MPCs and P2-MSCs were washed in MACSQuant™ Running Buffer (Miltenyi Biotech, Bergisch Gladbach, Germany) and stained with anti-CD11c VioBlue®, anti-CD18 APC, anti-CD31 PE, anti-CD34 VioBlue®, anti-CD45 APC-Vio770, anti-CD73 PE, anti-CD90 FITC, anti-CD133 APC, anti-CD146 FITC, HLA-DR VioBlue® (Miltenyi Biotech), anti-STRO-1 FITC, and CD144 PE (Biolegend, San Diego, CA, USA). Samples were acquired by MACSQuant® Flow Cytometer and analyzed by MACSQuantify® Software (Miltenyi Biotech).

#### Tricolor immunofluorescence

Freshly isolated MPCs, P2-MSCs, and HUVECs were plated in two-well Lab-Tek™ Permanox chamber slides (Thermo Scientific, Rochester, NY, USA). Slides were fixed for 15 min in 4% paraformaldehyde at room temperature and subsequently permeabilized with 0.5% Triton X-100 for 30 min. Immunofluorescence was carried out using mouse monoclonal anti-human Nestin (Abcam, Cambridge, UK) and rabbit polyclonal anti-human von Willebrand factor antibodies (Abcam). Positive stain was revealed by the goat anti-mouse SFX kit (Thermo Scientific), according to the manufacturer’s instructions using AlexaFluor®-488 anti-mouse IgG and AlexaFluor®-555 anti-rabbit IgG (Thermo Scientific). F-Actin was detected by AlexaFluor®-555 Phalloidin (Thermo Scientific). Slides were mounted in Prolong® Gold antifade reagent with 4′,6-diamidino-2-phenylindole (DAPI; Thermo Scientific) for nuclei detection. Pictures were taken and combined using a standard fluorescence DMR Leica microscope (Leica, Wetzlar, Germany) equipped with Leica CW4000 image software (Leica).

#### Mesengenic terminal differentiation

P2-MSCs cultured in chamber slides were induced to terminal differentiation into adipocytes using StemMACS® AdipoDiff Medium (Miltenyi Biotech) or into osteocytes by StemMACS® OsteoDiff Medium (Miltenyi Biotech). Media were refreshed every 48 h and cultures were maintained for 21 days.

To detect lipid droplet accumulation, the medium was removed, wells were washed twice with prewarmed D-PBS, and cells were incubated in 200 nM Nile Red (Thermo Scientific) for 10 min at 37 °C in the dark. Calcium deposits were revealed by OsteoImage™ Mineralization assay kit (Lonza) according to the manufacturer’s instructions. Pictures were taken using an inverted fluorescence DM IRB Leica microscope (Leica), equipped with LAS image acquisition software (Leica).

### Gene expression analysis

Freshly isolated MPCs, P2-MSCs, and HUVECs were processed for gene expression analysis of endothelial-associated genes (*PECAM*, *vWF*, *CDH5*, *KDR*, *TEK, TIE1*, *DLL4*, and *JAG1*), mesenchymal/pericyte-associated genes (*ACTA2*, *DES*, *CSPG4*, *RUNX2*, *TEM1*, *MCAM*, *RGS5*, and *LEPR*), MPC-related genes (*NES*, *OCT-4A*, *SPP1*, *NANOG*, *GP130*, *LIFR*, and *RANK*), and cytokines (*SCF*, *ANGPT1*, *ANGPT2*, *PDGFA*, *PDGFB*, and *RANKL*). Total RNA was extracted using the RNeasy Micro Kit (Qiagen GmbH, Hilden, Germany) according to the manufacturer’s instructions. RNA samples (100 ng) were retro-transcribed using a QuantiTect® Reverse Transcription Kit (Qiagen) and 2 μl samples of 10-fold cDNA dilutions were amplified by quantitative real-time PCR (qRT-PCR), using the iCycler-iQ5 Optical System (Bio-Rad, Hercules CA, USA) and SsoAdvancedSYBR Green SuperMix (Bio-Rad). Samples were run in duplicate. All primer pairs (see Additional file 1) were from Sigma-Aldrich. Relative quantitative analysis was performed following the 2^–ΔΔCt^ Livak method [12]. Normalization was performed using *RPL13A* and *ACTB* housekeeping genes. Hierarchical clustering analysis was performed by applying HeatmapGenerator 5 software [13]. Values were reported as mean of normalized fold expression ± SEM. Statistical analysis was carried out by two-tailed *t* test applying the Mann–Whitney test.

### In-vitro evaluation of angiogenic potential

#### Acetylated-low density lipoprotein uptake

Freshly isolated MPCs, P2-MSCs, and HUVECs were seeded at confluence in six-well plates and left to attach overnight. Cultures were then incubated for 4 h at 37 °C with 5 μg/ml AlexaFluor488®-conjugated acetylated-low density lipoprotein (Ac-LDL; Thermo Scientific) in DMEM/1% BSA. Cells were washed twice and pictures taken as already described using an inverted fluorescence microscope. Binary images were obtained by Qwin® Image Analysis Software (Leica) to quantify fluorescent areas.

#### Static transendothelial migration assay

The migration assay was performed in a modified Boyden chamber system [14] by colorimetric QCM™ Transendothelial Migration Assay (Millipore, Billerica, MA, USA), according to the manufacturer’s instructions. Briefly, 1 × 10^5^ HUVECs were seeded in a monolayer on 24-well fibronectin-coated 8-μm culture inserts and activated with 20 ng/ml recombinant human TNF-α for 24 h. In parallel, freshly isolated MPCs and P2-MSCs were starved in DMEM under constant agitation. After 24 h, 1 × 10^5^ cells were seeded on top of the HUVEC monolayers and culture inserts hung in 24-well plates containing DMEM, DMEM supplemented with 10% fetal bovine serum (FBS), and DMEM enriched with 50 μg/ml of SDF-1β, respectively. HUVECs containing inserts only were used as negative controls. After 24 h of incubation, nonmigrating cells and HUVECs were removed by swabbing the insert top surface, whereas cells which had migrated to the external surface of the inserts were stained with a colorimetric solution for 15 min at room temperature. Quantification was performed by measuring the absorbance at 570 nm after solubilization of the dye with extraction solution.

#### Capillary-like tube formation assay

Twenty-four-well culture plates were set up with 300 μl aliquots of Matrigel® (BD Bioscience, CA, USA) and incubated for 30 min at 37 °C to allow polymerization. Freshly isolated MPCs, P2-MSCs, and HUVECs were seeded over Matrigel® thick layers at 3 × 10^3^ or 5 × 10^3^ cells/well and cultured in EGM-2 medium (Lonza) at 37 °C, 5% CO_2_. After 48 h, phase-contrast microphotographs were taken and processed for image analysis to measure tube lengths and lumen areas.

#### Assessment of sprouting angiogenesis in 3D culture

Six 3D spheroid sets were generated by the hanging drop method [15, 16]. Drops (20 μl) of freshly isolated MPC, P2-MSC, and HUVEC suspensions (1.5 × 10^5^ cells/drop) were laid on the inner surface of a Petri dish lid. To prevent hanging drop evaporation, the lids were used to recap Petri dishes containing PBS and incubated overnight at 37 °C in 5% CO_2_ for cell aggregation. Spheroids were gently applied onto the Matrigel® thick gel layer and cultured in EGM-2 medium. Sprouting was evaluated after 24 h (sets of three spheroids) and after 7 days (sets of three spheroids) by measuring the distance between the last invading cell and the spheroid edge. Measurements were performed independently by three operators and mean values recorded. After image analysis, 3D cultures were refrigerated at 4 °C for 2 h to allow Matrigel® melting and washed in pre-refrigerated D-PBS. Spheroids were harvested by centrifugation at 300 × *g*. Sets of five spheroids were processed for RNA extraction and gene expression analysis of *NES*, *SPP1*, *PECAM1*, *TEM1*, *TIE1*, *TEK*, *KDR*, *JAG1*, *vWF*, *DLL4*, *PDPN*, *PROX-1*, and *FLT-4*. The additional sets of three spheroids were enzymatically dissociated by TryPLE Select® (Thermo Scientific) to obtain single cell suspensions for the capillary tube-like formation assay.

### Ex-ovo chicken chorioallantoic membrane assay

Fertilized White Leghorn chicken eggs were incubated for 72 h at 37 °C in a humidified rotary incubator (Covatutto 108; Novital, Treviso, Italy). At day 3 of embryonic development, eggs were cracked under sterile conditions and embryos gently laid into plastic weighting boats (Sigma-Aldrich) covered with square Petri dish lids (Sarstedt, Nümbrecht, Germany). Embryos were maintained for an additional 5 days at 37 °C in a stationary humidified incubator.

At day 8 post fertilization, 20 μl drops of freshly isolated MPC, P2-MSC, and HUVEC suspensions (1.5 × 10^5^ cells/drop) were laid on the inner surface of a Petri dish lid and coated with 30 μl of Geltrex™ LDEV-Free Reduced Growth Factor Basement Membrane Matrix (Thermo Scientific). Constructs were allowed to solidify at 37 °C for at least 2 h. Geltrex™ droplets (50 μl) were set up as “no cell” negative controls. Constructs were grafted onto the embryo chorioallantoic membrane (CAM), carefully avoiding pre-existing blood vessels, and silicone O-rings were placed around individual on-plants to facilitate localization. Embryos were further incubated and pictures were taken at days 10 and 11 using a stereomicroscope (SZ40; Olympus, Tokyo, Japan) equipped with a digital camera (Digital SLR Camera D200; Nikon, Tokyo, Japan). Pictures were then processed by the WimCAM image analyses web-based service (Onimagin Technologies, Spain) to quantify angiogenesis. Data on total vessel network length, total branching points, total segments, and mean segment length were recorded and reported as mean ± SEM. Statistical analysis was performed by applying a one-way ANOVA test and Dunnett’s post test.

On-plants and surrounding areas were sectioned and fixed in buffered 4% formalin solution for 24 h. After paraffin embedding, serial 5-μm sections were processed for hematoxylin/eosin stain. In addition, anti-human leukocyte antigen HLA Class 1 ABC antibody (AbCam) was applied in order to reveal human cells. The SuperPicture™ 3rd Gen IHC Detection Kit (Thermo Scientific) was applied according to the manufacturer’s instructions to reveal positivity, and sections were counterstained with Gill’s n.3 hematoxylin (Sigma-Aldrich). Slides were observed and photographed using a DMR Leica standard microscope (Leica). Immunofluorescent detection of human cells was also performed applying goat anti-mouse IgG secondary antibody conjugated to Alexa Fluor® 488 dyes (1-h incubation at room temperature), respectively. Nuclei were counterstained with TO-PRO 3 (15-min incubation) and slides were mounted with ProLong Diamond (Thermo Scientific). Slides were incubated previously in D-PBS/0.3 M Glycine, as an enhancer, for 20 min and blocked in D-PBS containing 10%(v/v) normal goat serum (AbCam) and 0.5% (w/v) BSA (Sigma Aldrich) for 1 h. A negative control was performed in each case by omitting the primary antibody. Imaging was performed on the SP8 confocal microscope (Leica) and tubular structures were evaluated by Z-stack (steps of 0.35 μm) with 3D reconstruction.

## Results

### MPC isolation, characterization, and mesengenic differentiation

**Fig. 1:**
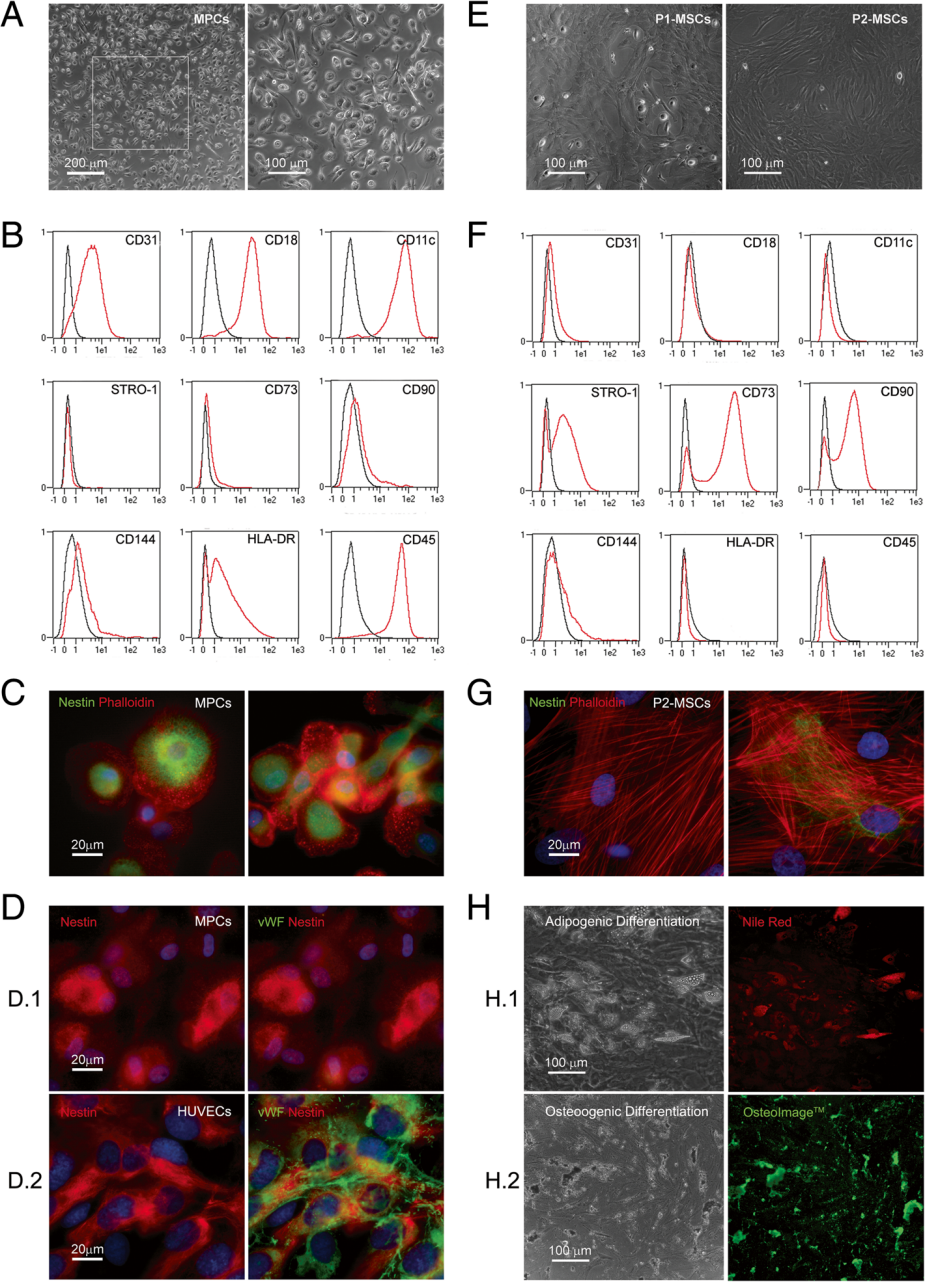
Cell culture characterization. Phase contrast microscopy of 6-day BM-MNCs in selective culture conditions showed adherent cells characterized by distinctive MPC morphology with round fried egg-shape and sporadic polar elongation (**a**). Flow cytometry showed homogeneous expression of MPC-related markers CD31, CD18, and CD11c and lack of MSC markers STRO-1, CD73, and CD90 (**b**). Immunofluorescence revealed F-actin typical podosome-like distribution (*red*) and intense positive stain for nestin (*green*) (**c**). Nuclei visualization was performed by DAPI staining (*blue*). No difference in nestin expression (*red*) was found between MPCs (*d.1*) and HUVECs (*d.2*) while von Willebrand factor (*vWF*) was detected in HUVECs only (*green*) (**d**). One-week MPC mesengenic differentiation (P1-MSCs) produced mixed cultures of flattened elongated multibranched cells with residual highly rifrangent MPCs. Subculturing P1-MSCs for a further week in MesenPRO® RS Medium led to monomorphic cultures of confluent fibroblastoid spindle-shaped cells (P2-MSCs) (**e**). Flow cytometry revealed a standard MSC phenotype for P2-MSCs (**f**). Immunofluorescence showed F-actin reorganization in stress fibers (*red*) and loss of nestin, occasionally expressed by few residual multibranched cells (*green*) (**g**). P2-MSCs were also able to differentiate selectively into adipocytes, as revealed by intracellular lipid droplet accumulation (*red* in *h.1*) or osteocytes featuring intense extracellular calcium deposition (*green* in *h.2*) (**h**). *HUVEC* human umbilical vein endothelial cell, *MPC* mesangiogenic progenitor cell, *MSC* mesenchymal stromal cell (Color figure online)

MPCs were consistently isolated from human bone marrow in selective culture conditions [5], with an average yield of 1.2 ± 0.7% (*n* = 12). Phase contrast microscopy confirmed MPC distinctive morphology, characterized by a round, highly rifrangent fried-egg shape (Fig. 1a) [1]. Over 90% of cells (93.6 ± 5.9%, *n* = 12) expressed MPC-associated markers CD31 (PECAM), CD18 (Integrin β2), and CD11c (Integrin αX) while lacking vascular endothelial (VE)-cadherin (CD144) as well as MSC-associated markers STRO-1, CD73, and CD90 (Fig. 1b). Possible contamination by endothelial and hemopoietic progenitors was excluded by flow cytometry, which did not detect either CD34-positive or CD133-positive events (<0.1% of total events, data not shown). Pericyte contamination was excluded by the absence of CD146-positive cells (<0.2% of total events, data not shown). Immunofluorescence analysis showed the characteristic intense positive stain for nestin and the dotted F-actin organization in podosome-like structures (Fig. 1c) [4]. Endothelial cell marker von Willebrand factor (vWF), evident in control HUVECs and often organized in strings and Weibel-Palade bodies (Fig. 1d.2), was not detected in MPC cultures (Fig. 1d.1), confirming the absence of mature endothelial cells in these cell preparations.

MPCs reproducibly differentiated toward the mesengenic lineage through the two-step process we described previously [6]. Proliferating, flattened, multibranched cells were detectable, alongside residual undifferentiated MPCs, after 1 week of culture in MesenPRO® RS medium (P1-MSCs). A second confluence was reached after a further 1 week of selective culture (P2-MSCs). Cells showed MSC-like spindle-shaped morphology (Fig. 1e) and expressed mesenchymal markers STRO-1, CD73, and CD90 while MPC-associated markers became undetectable (Fig. 1f). P2-MSCs revealed F-actin reorganization in typical stress fibers while nestin expression was confined to a few rare cells (Fig. 1g). Adipogenic and osteogenic differentiation potential was also assayed. After 2 weeks under specific differentiating conditions, P2-MSCs showed the ability to terminally differentiate into adipocytes, as revealed by lipid droplet intracellular accumulation (red in Fig. 1h.1), or into osteocytes, evidenced by extracellular mineralized matrix deposition (green in Fig. 1h.2). In parallel, P2-MSC cultures maintained in MesenPRO® RS medium (undifferentiated controls) were negative to Nile Red and OsteoImage™ (data not shown).

### Gene expression analysis

**Fig. 2:**
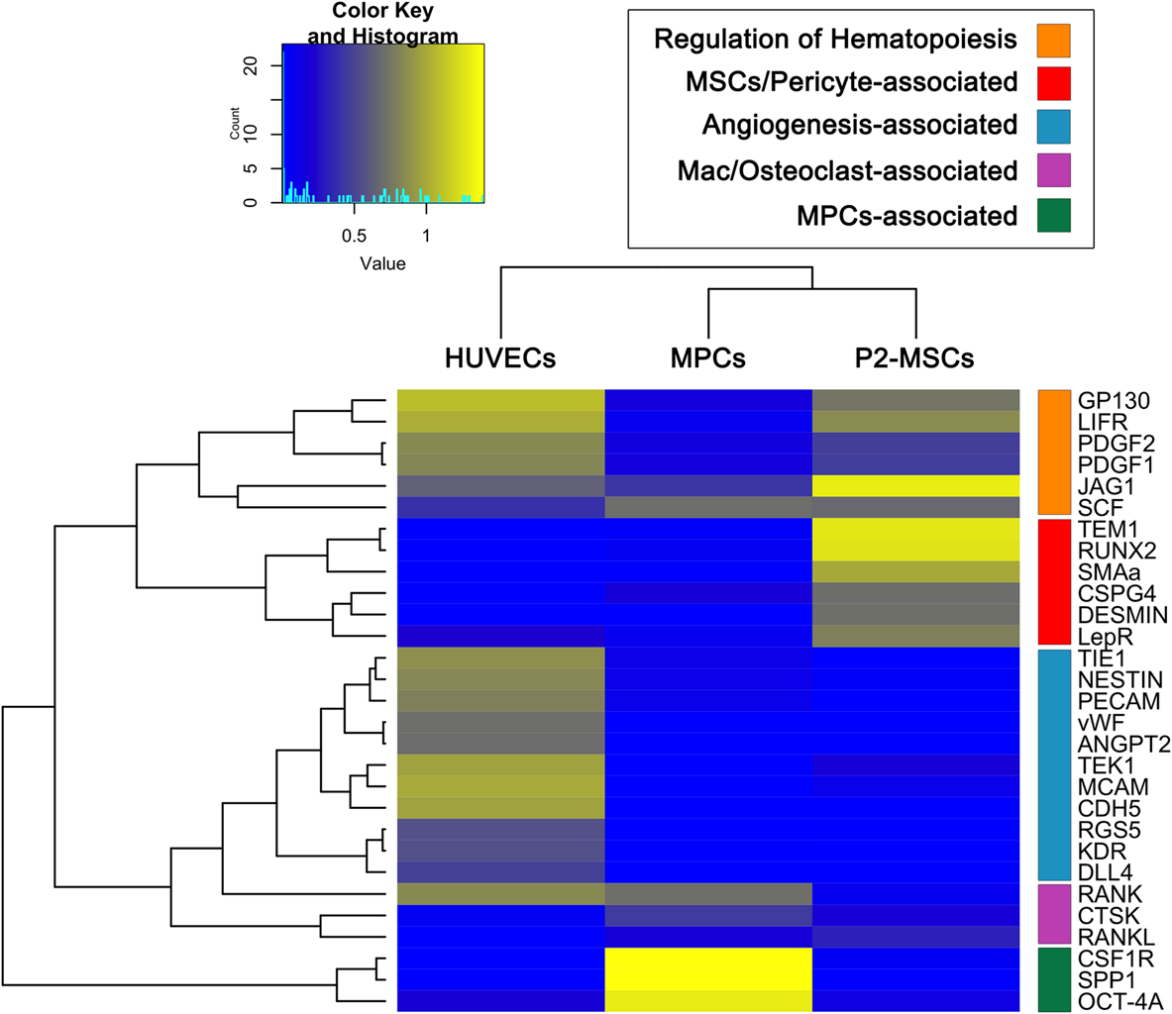
Hierarchical clustering analysis. Expression of the selected 29 genes reveals five major clusters and three of them were specific for each cell population. HUVECs exclusively expressed angiogenic-associated cluster (*pale blue*). A cluster of three genes (*CSF1R*, *SPP1*, and *OCT-4A*; *green*) was specific for MPCs and silenced after mesengenic differentiation in P2-MSCs, which upregulated the MSC/pericyte-associated cluster (*red*). As expected, MPCs were more hierarchically closely associated with P2-MSCs. *HUVEC* human umbilical vein endothelial cell, *MPC* mesangiogenic progenitor cell, *MSC* mesenchymal stromal cell (Color figure online)

**Fig. 3:**
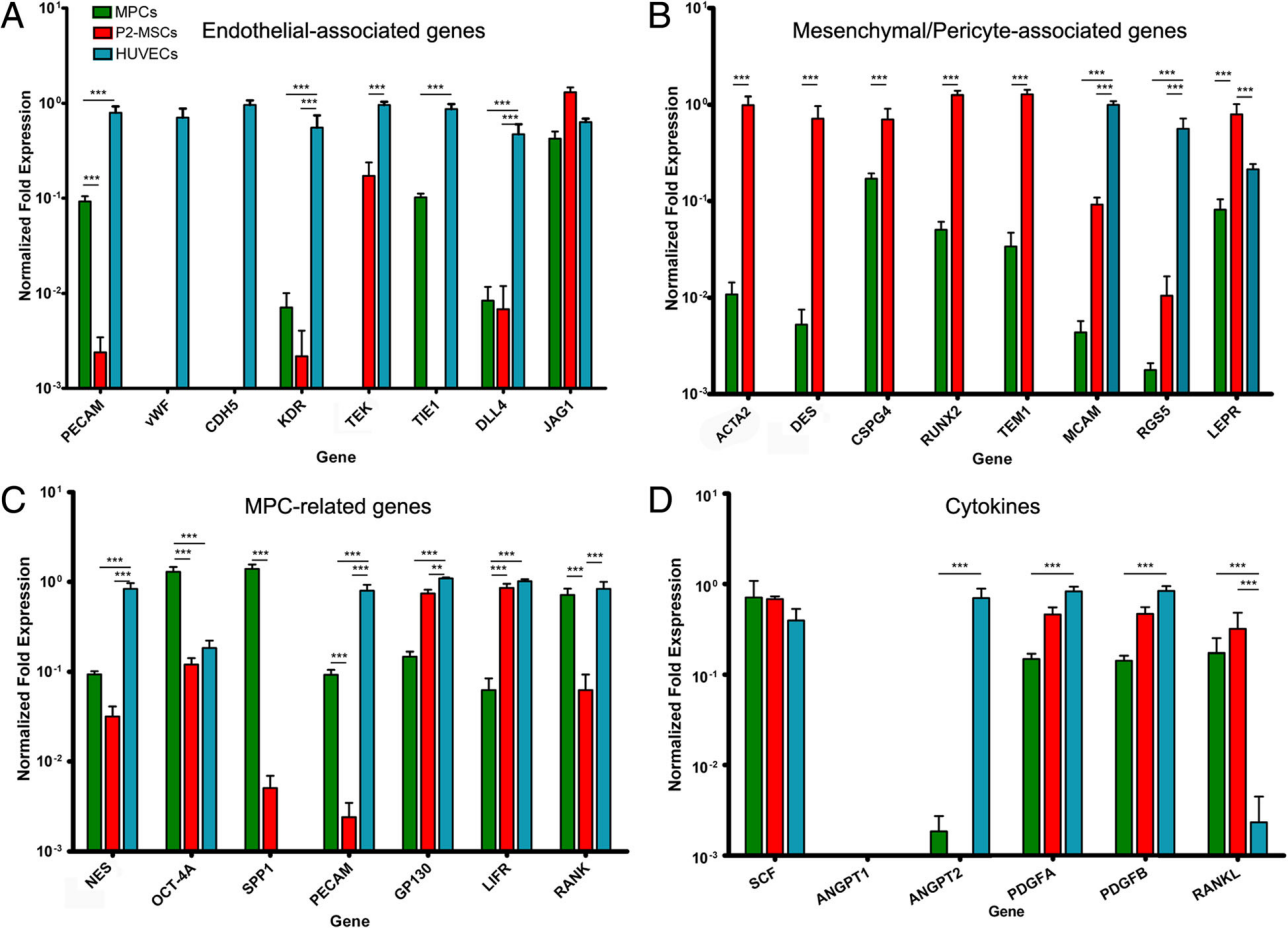
Gene expression analysis. Endothelial-associated genes (**a**) were constitutively expressed by HUVECs (*blue*). *PECAM* and *TIE1* were highly expressed in MPCs (*green*) and silenced during mesengenic differentiation. Conversely, P2-MSCs (*red*) activated *TEK* expression. MPC mesengenic differentiation was accompanied by mesenchymal/pericyte-associated gene (**b**) upregulation and significant reduction of MPC-related genes *NES*, *OCT-4A*, and *SPP1* (**c**). Cytokine gene expression was not significantly modified following mesengenic induction (**d**). Differences over 1 log were evidenced for *ANGPT2* and *RANKL* when comparing MPCs/P2-MSCs with HUVECs. **p < 0.01, ***p < 0.001. *HUVEC* human umbilical vein endothelial cell, *MPC* mesangiogenic progenitor cell, *MSC* mesenchymal stromal cell (Color figure online)

Freshly isolated MPCs, P2-MSCs, and HUVECs were analyzed for expression of 29 genes involved in bone marrow homeostasis. Hierarchical clustering analysis identified five major clusters differentially expressed in the three cell preparations. The cluster expression patterns were peculiar for each cell population and revealed MPCs more closely associated with P2-MSCs in the sample hierarchy (Fig. 2), as expected considering that P2-MSCs has been obtained by MPC mesengenic differentiation. Statistical analysis of gene expression was performed normalizing mRNA to the levels detected in HUVECs’ (10^0^ reference value; blue bars in Fig. 3a).

Endothelial-associated genes (*PECAM*, *vWF*, *CDH5*, *KDR*, *TEK, TIE1*, *DLL4*, and *JAG1*) showed constitutive high expression in HUVECs. MPC immunophenotype was confirmed by marked expression of *PECAM* (CD31) (9.3 ± 1.2 × 10^−2^, *n* = 12, *p* < 0.001) and lack of *vWF* and *CDH5* (CD144). MPCs revealed very low levels of *KDR* (7.0 ± 3.1 × 10^−3^, *n* = 12, *p* < 0.001) and *DLL4* (8.2 ± 3.2 × 10^−3^, *n* = 12, *p* < 0.001) while *TIE1* expression was only 1 log lower (1.0 ± 0.1 × 10^−1^, *n* = 12, *p* < 0.001) than in HUVECs. *TEK* was not expressed while *JAG1* levels were similar to HUVEC levels (green bars in Fig. 3a).

P2-MSCs showed a consistent mesengenic pattern of gene expression, including downregulation of *PECAM* (1.8 ± 0.2 × 10^−3^, *n* = 10, *p* < 0.001), silencing of *TIE1*, and triggering of *TEK* (1.7 ± 0.6 × 10^−1^, *n* = 10, *p* < 0.001; red bars in Fig. 3a). A comprehensive analysis of mesenchymal/pericyte-associated genes showed selective upregulation in P2-MSCs as compared with MPCs. In particular, *CSPG4*, *RUNX2*, *TEM1*, *MCAM*, *RGS5*, and *LEPR* expression was over 1 log higher while *ACTA2* and *DES* were over 2 logs higher in P2-MSCs (*p* < 0.001; Fig. 3b). Most of the genes were not expressed in HUVECs, with the exception of *MCAM*, *RGS5*, and *LEPR*. Expression of the *MCAM* and *RGS5* was even higher in HUVECs than in P2-MSCs.

MPC-related gene modulation following mesengenic induction was significant for *OCT-4A* and *RANK* (around 1 log lower in P2-MSCs as compared with freshly isolated MPCs, *p* < 0.001), as well as for *SPP1* (over 2 logs reduction, *p* < 0.001). *GP130* and *LIFR* were mildly upregulated (*p* < 0.001) while *NES* reduction was not statistically significant (Fig. 3c).

Cytokine gene expression was not significantly modified following mesengenic induction (Fig. 3d). Differences over 1 log were evidenced for *ANGPT2* expressed by HUVECs only and for *RANKL* expressed by MPCs and P2-MSCs (*p* < 0.001; Fig. 3d).

### In-vitro angiogenesis-related assays

**Fig. 4:**
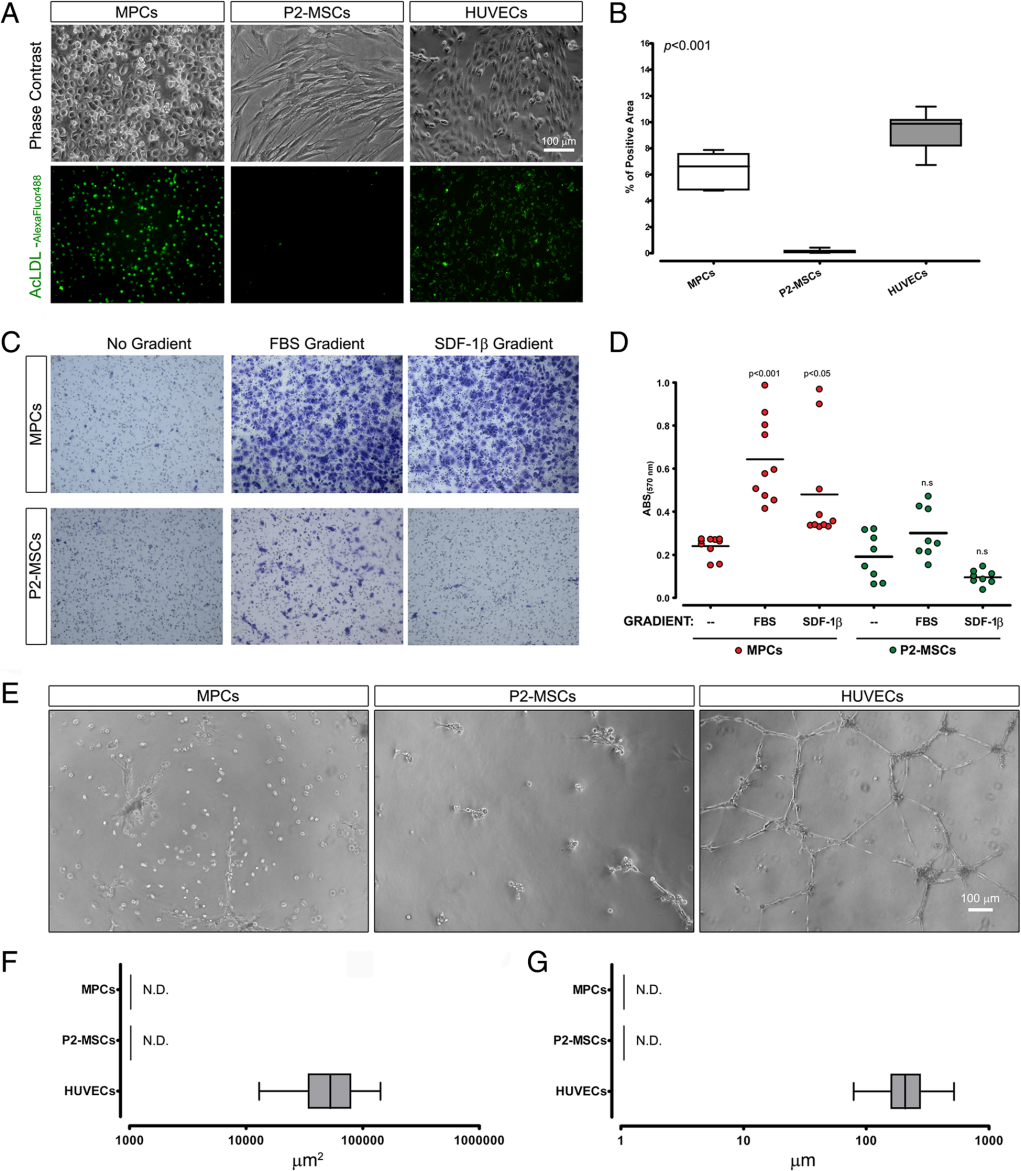
In-vitro angiogenesis-related assays. Freshly isolated MPCs as well as HUVECs revealed substantial Ac-LDL uptake, showing green fluorescence in most adherent cells. P2-MSCs showed no fluorescence (**a**). Quantitative evaluation (percentage of fluorescent areas) confirmed the observation (**b**). MPC transendothelial migration ability was reported in the presence of either FBS gradient or SDF-1β chemoattraction with a number of migrated cells on the outer surface of culture inserts (**c**, *blue–violet*). Conversely, few migrating P2-MSCs were reported exclusively under FBS gradient and only in part of the samples, compromising statistical significance (**d**) (“n.s.”, *p* > 0.05). Neither MPCs nor P2-MSCs were able to form CLS in capillary-like tube formation assay (**e**, **f**, **g**). *Ac-LDL* acetylated-low density lipoprotein, *FBS* fetal bovine serum, *HUVEC* human umbilical vein endothelial cell, *MPC* mesangiogenic progenitor cell, *MSC* mesenchymal stromal cell, *N.D.* not detected, *SDF-1β* stromal cell-derived factor 1 beta (Color figure online)

Freshly isolated MPCs revealed substantial Ac-LDL uptake, which was totally absent in P2-MSCs (Fig. 4a). The significantly lower percentage of fluorescent areas per field in MPCs (6.3 ± 1.3%, *n* = 30, *p* < 0.001) as compared with HUVECs (9.3 ± 1.5%, *n* = 15; Fig. 4b) appears to be related to a different distribution in the intercellular compartments rather than to a lower percentage of fluorescent cells (green in Fig. 4a).

Static transendothelial migration assay showed the ability of MPCs to migrate through the activated endothelial layer in the presence of either FBS gradient (*p* < 0.001) or SDF-1β chemoattraction (*p* < 0.05). Transendothelial migration was suppressed in P2-MSCs (*p* < 0.05; Fig. 4c, d).

In the capillary-like tube formation assay, HUVECs rapidly and efficiently formed networks of CLS with lumen areas around 60,000 μm^2^ (59,789 ± 32,465 μm^2^, *n* = 160; Fig. 4e, f) and branching lengths of about 200 μm (218.4 ± 80.4 μm, *n* = 150; Fig. 4e, g). Conversely, neither MPCs nor P2-MSCs were able to form CLS (Fig. 4e), even after prolonged culture times (data not shown).

### In-vitro assessment of sprouting angiogenesis

**Fig. 5:**
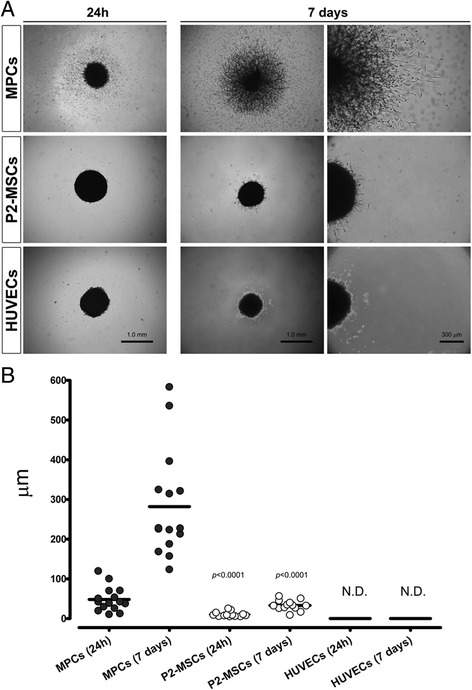
Sprouting from 3D spheroids. Substantial differences among cell populations were found in culturing spheroids on Matrigel®. A front of gel invasion was rapidly (24 h) detected in MPC-derived spheroids while spheroids from P2-MSCs and HUVECs showed more compact structures with sharper edges (**a**). After 1 week, MPC spheroids showed loose network structures and sprouting over 250 μm from the edge. Sprouting from P2-MSC spheroids was definitely reduced with only few cells detected within a shorter (35 μm) distance from the edge. Conversely, HUVECs showed no invading capacity (**b**). *HUVEC* human umbilical vein endothelial cell, *MPC* mesangiogenic progenitor cell, *MSC* mesenchymal stromal cell, *N.D.* not detected

3D spheroids from MPCs and P2-MSCs were sufficiently compact to be manipulated easily. In contrast, spheroids generated from HUVECs showed worse mechanical quality, making them very difficult to handle and to apply on Matrigel®. After 24 h of culture on Matrigel®, some invading cells started to appear within 50 μm (48.2 ± 31.0 μm, *n* = 12) from the edge of MPC spheroids. Conversely, the edge of P2-MSC spheroids was very sharp and few invading cells appeared in only three out of 20 spheroids, within a significantly shorter distance (10.6 ± 6.4 μm, *n* = 10, *p* < 0.0001) (Fig. 5). Prolonged culture time (7 days) revealed evident sprouting angiogenesis in all directions from MPC spheroids, with distances from the edge estimated between 100 and 600 μm (282.1 ± 134.4 μm, *n* = 15). MPCs lost their sprouting ability once terminally differentiated into P2-MSCs with rare invading cells within a very short distance (33.1 ± 13.0 μm, *n* = 10, *p* < 0.0001). In our experimental setting, sprouting activity from HUVEC spheroids was not detected (N.D., *n* = 6).

**Fig. 6:**
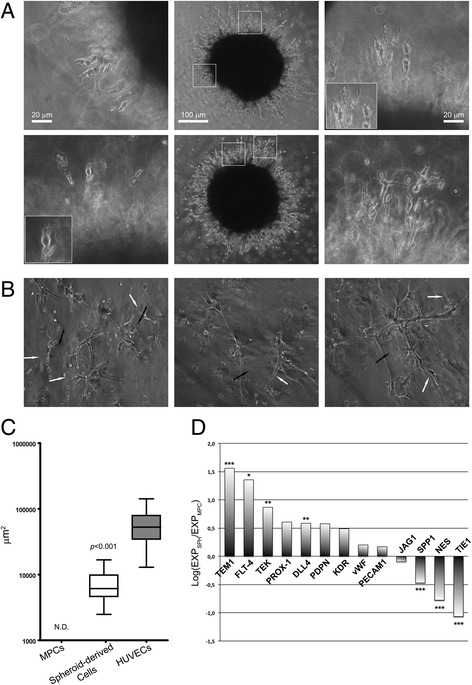
Sprouting cells from MPC-derived spheroids acquire endothelial tip cell-like features. Analyzing the invading front of MPC spheroids, most cells at the distal ends of invading branches showed the bipolar morphology characteristic of endothelial “tip cells” (**a**). Cells harvested from enzymatic digestion of sprouted MPC spheroids acquired the ability to form CLS with numerous closed pseudo-lumens (**b**, *black arrows*; three representative samples displayed), although showing smaller lumen mean areas than HUVECs (**c**). Many “tip cell-like” invading cells were still detectable after MPC spheroid enzymatic digestion (**b**, *white arrows*). Gene expression analysis confirmed MPC-related gene (*SPP1* and *NES*) downregulation together with activation of genes involved in angiogenic and lymphoangiogenic sprouting as *TEM1*, *FLT-4*, *PROX-1*, and *DLL4* (**d**). *p < 0.05, **p < 0.01, ***p < 0.001. *HUVEC* human umbilical vein endothelial cell, *MPC* mesangiogenic progenitor cell, *N.D.* not detected


Analyzing the invading front of MPC spheroids after 24 h of 3D culture in Matrigel®, we detected a high number of bipolar cells characterized by a highly branched distal end and a hemispheric shaped proximal end (Fig. 6a, Additional file 2). This peculiar morphology is reminiscent of typical activated endothelial “tip cells” [17, 18]. Cells harvested from enzymatic digestion of sprouted MPC spheroids revealed the ability to form CLS even though at lower efficiency than HUVECs, in terms of closed tube number per field and lumen areas (7,393 ± 702 μm^2^, *n* = 29 vs 59,740 ± 4459 μm^2^, *n* = 53, *p* < 0.001; Fig. 6b, c). Many “tip cell-like” invading cells were still detectable after MPC spheroid enzymatic digestion (white arrows in Fig. 6b). In contrast, they were undetected in the HUVEC standard Matrigel® morphogenic assay (Fig. 4f).

Additional file 2: Is a video showing invading endothelial “tip-like” cell sprouting from MPC-derived 3D spheroids. Sprouting fronts were characterized by the presence of peculiar cells showing bipolar morphology with highly branched distal ends (*white arrows*) juxtaposed to hemispheric shaped proximal ends. Video shows focal plans scrolling on the *Z* axis. (MP4 676 kb)


Gene expression analysis of MPC spheroid-derived cells at 7 days revealed significant upregulation of *TEM1* (*p* < 0.001), *FLT-4* (*p* < 0.05), *DLL4* (*p* < 0.01), and *TEK* (*p* < 0.01) as compared with undifferentiated MPCs. The *PDPN*, *PROX-1*, and *KDR* media fold expression ratio was consistently positive, demonstrating upregulation during sprouting. Nonetheless, the variability of those expression data did not allow obtaining a statistical significance. MPC-related genes *NES* and *SPP1* together with *TIE1* were significantly downregulated (*p* < 0.001; Fig. 6d). *PECAM1* and *JAG1* expression detected in MPC was not affected during sprouting, while *vWF* expression was not induced during this step of MPC differentiation.

### Ex-ovo CAM assay

**Fig. 7:**
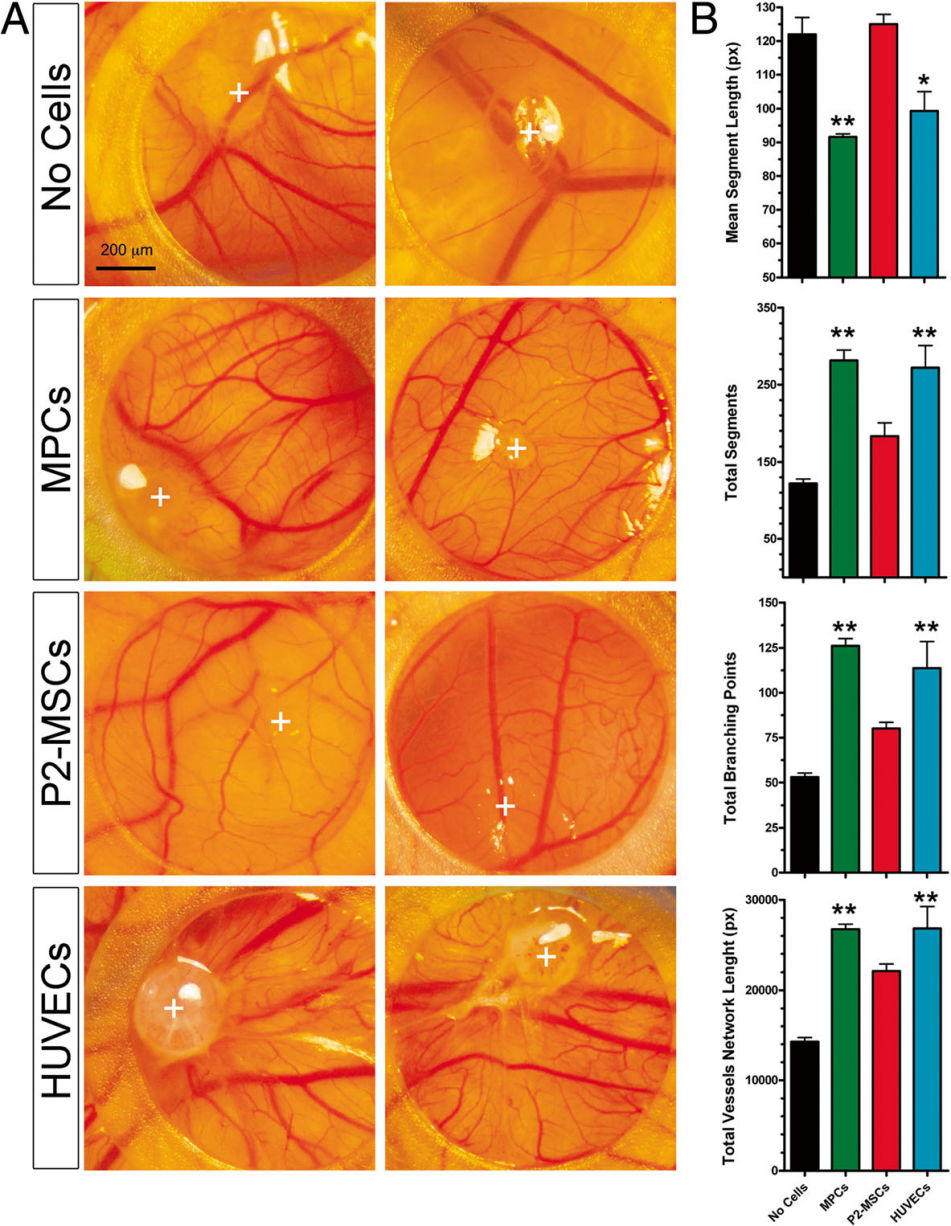
Ex-ovo CAM assay. Alterations of the vascular network were analyzed in 8-day chicken embryo CAM, 72 h after grafting Geltrex™ droplets containing MPCs, P2-MSCs, and HUVECs. MPC and HUVEC grafts stimulated the formation of a more complex vessel network as revealed by numerous afferent capillaries, while P2-MSCs did not show any significant effect with respect to “no cell” negative controls. Two representative samples for each cell type are displayed (**a**). *White crosses*, implant graft sites. Quantification of the capillary network surrounding the on-plants confirmed the increased complexity in the tests applying MPCs (*green*) or HUVECs (*pale blue*), in terms of total network length, branching points, number of segments, and mean segment length, with respect to “no cell” control (*black*) or MSCs (*red*) (**b**). *p < 0.05, **p < 0.01. *HUVEC* human umbilical vein endothelial cell, *MPC* mesangiogenic progenitor cell, *MSC* mesenchymal stromal cell (Color figure online)

Alterations of the vascular network were analyzed in 8-day chicken embryo CAM by grafting gelified Geltrex™ droplets containing MPCs, P2-MSCs, and HUVECs. MPC grafts effectively produced increased in-vivo neovessel formation as revealed by numerous afferent capillaries, and significantly higher vessel network length (26,742.0 ± 569.9 px, *p* < 0.01, *n* = 5) with respect to Geltrex™ alone (14,286.0 ± 461.9 px, *n* = 5). Moreover, the MPC-induced vessel network showed an increased number of branching points (126.0 ± 4.2 vs 53.0 ± 2.3, *p* < 0.01, *n* = 5), increased total number of segments (281.0 ± 13.5 vs 122.0 ± 5.8, *p* < 0.01, *n* = 5), and consequently reduced mean segment length (91.70 ± 0.88 vs 122.12 ± 5.08 px, *p* < 0.01, *n* = 5). P2-MSC grafts showed no significant effect, with close resemblance to “no cell” negative controls. HUVEC grafts stimulated the formation of a more complex vessel network with respect to control, and similar to MPCs (Fig. 7).

**Fig. 8:**
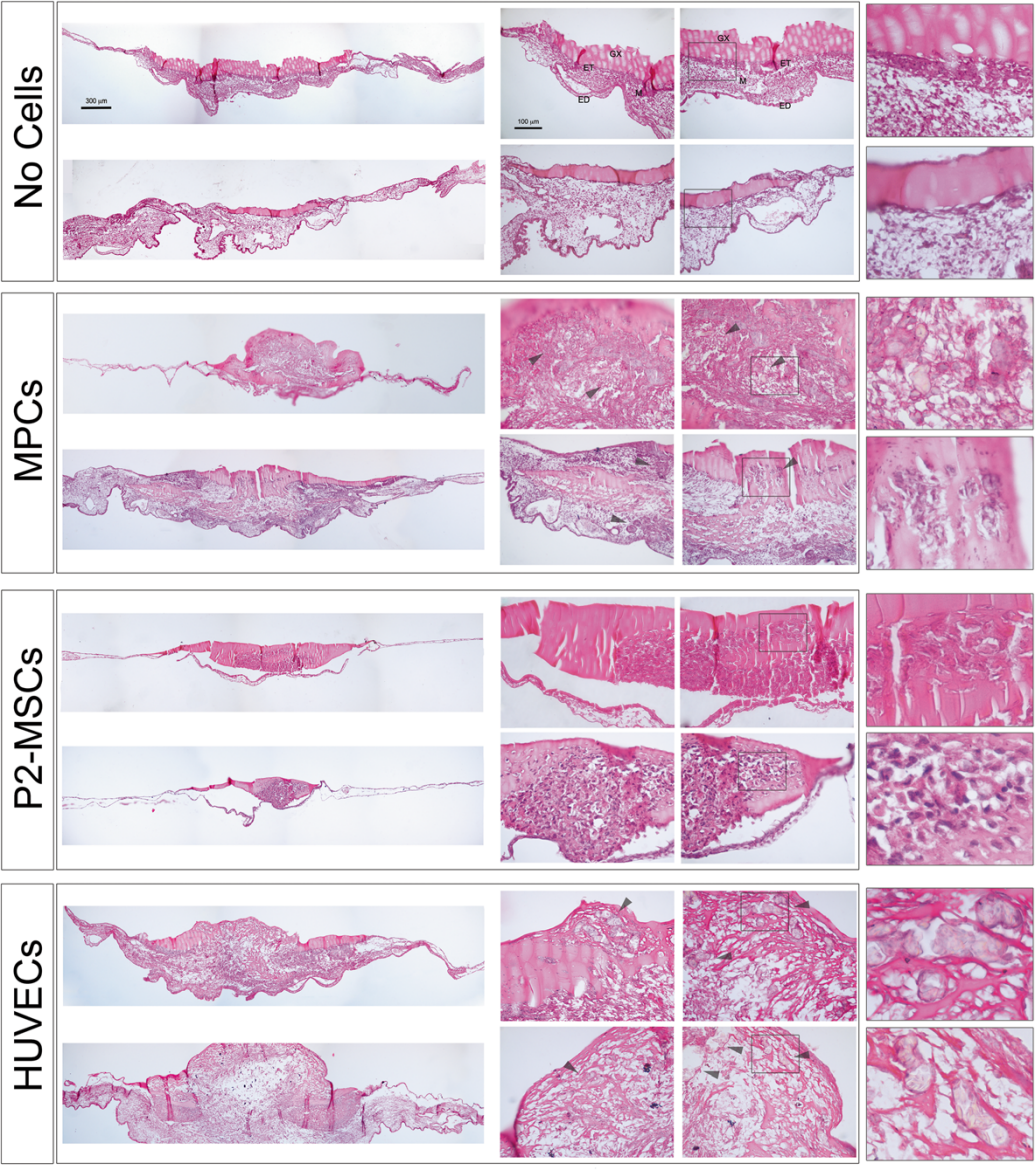
Histological examination of chicken embryo CAM grafts. Application of “no cell” grafts did not alter CAM morphology, allowing clear identification of the three-layer structure formed by endoderm (*ED*), mesoderm (*M*), and ectoderm (*ET*). MPC grafts showed extended areas of Geltrex™ degradation and intense tissue remodeling, with an increased number of newly formed microvessels (*arrows*, exploded frame). P2-MSC implants maintained their compact structure and did not interact with CAM; no increment of vascularization was detected. HUVEC grafts revealed Geltrex™ degradation, CAM remodeling, and increased vascularization. Two representative samples for each cell type are displayed. *HUVEC* human umbilical vein endothelial cell, *MPC* mesangiogenic progenitor cell, *MSC* mesenchymal stromal cell


To further characterize the neovessel formation process, CAM histological analysis was carried out after 72 h from implants. “No cell” grafts did not alter the CAM morphology, allowing the unambiguous identification of endodermal, mesodermal, and ectodermal layers, with the latter maintaining its identity and structure despite close contact with the gelified matrix (pale pink in Fig. 8). Comparing grafted areas with distant CAM regions, the vascularization pattern was homogeneous throughout the mesodermal layer (data not shown). MPC grafts revealed extended areas of Geltrex™ degradation and intense tissue remodeling in the proximity of the implants. In particular, the CAM structure and spatial separation among graft, ectoderm, and mesoderm was lost; only the ectodermal layer remained unaltered and numerous newly formed capillaries were easily detectable (often organized in condensed groups) in the remodeling regions and in the lacunas generated by Geltrex™ degradation (arrows in Fig. 8). Immunohistochemistry for HLA confirmed that the remodeling regions involved the human derived cells (brown stained in Additional file 3) that were able to form hollow microtubules, resembling newly formed capillaries (see Additional file 4). Moreover, those foci of neovessel formation were also demonstrated to be of human origin for their positivity to hCD34 (red in Additional file 4 and Additional file 5). P2-MSC grafts showed a compact structure of Geltrex™ around cells with no sign of matrix degradation and CAM interaction (Fig. 8, Additional file 3). Indeed, the three-layer CAM morphology was maintained, no increased vascularization was detected, and implant attachment was impaired. HUVEC grafts induced substantial tissue and vascular remodeling, with histological features similar to those observed in MPC grafts. A consistent increase in chicken embryo-derived new vessels was detected around human cells. However, no human-derived tube-like structures were detected (Additional file 3). Moreover, unstructured human cell aggregates were negative for human CD34 antibody (Additional file 4).

Additional file 5: Is a video showing a 3D reconstruction of MPC-derived microtubules in chicken CAM on-plants. MPC on-plants showed a number of human cells, positive for HLA-ABC (*green*) and hCD34 (*red*), organized in hollow microtubules resembling newly formed capillaries. Nuclei shown in *blue*. (MP4 2900 kb)


## Discussion

In previous work we characterized bone marrow-derived MPCs for their capacity to differentiate through a two-step protocol into cells with endothelial phenotype, able to form CLS in Matrigel® morphogenic cultures [5]. In particular, freshly isolated MPCs were cultured in endothelial progenitor cell (EPC) EndoCult® Medium (using fibronectin-coated plates) [19] to an almost confluent monolayer of fibroblast-like cells partially expressing KDR/Flk-1 (CD309). A second step of angiogenic differentiation in VEGF-rich EGM-2 led to definite endothelial cell morphology and phenotype, characterized by CD31 expression and the ability to form CLS [1, 5]. The protocol low yield and additional MPC immunophenotypic characterization prompted us to further investigate MPC angiogenic differentiation.

In the present article we showed MPCs to possess most of the in-vitro properties usually attributed to endothelial progenitors or to cells capable of neo-angiogenesis [20], including Ac-LDL uptake, matrix degradation, and migration. These tissue invasion properties, involved in the early phases of neovascular formation from pre-existing vessels, are indicative of MPC early angiogenic potential. This hypothesis is corroborated by MPC marked 3D spheroid sprouting ability, which mimics the in-vivo tip/stalk cell modulation in vessel branching [21] characterized by *DLL4* induction of expression [22, 23]. Moreover, the consistent activation of *FLT-4* (VEGFR3) gene expression in sprouted MPCs sustains the idea of the “early stage” because this receptor has been detected in the embryonic veins immediately after differentiation from the angioblast. At this stage, cells are also potentially capable of triggering lymphoangiogenesis throughout activation of LYVE-1 in a VEGFR3^+^ subpopulation [24]. Notably, cells harvested from the enzymatic digestion of sprouted MPC-derived spheroids acquired the ability to form CLS, showing the achievement of a later stage of differentiation along the endothelial lineage. MPC angiogenic potential was substantiated in-vivo by the ability of MPCs to promote chicken CAM vascularization together with the demonstrated capability for participating in neovessel formation. The association between the in-vitro ability of sprouting from spheroids and the “early stage” as well as the association between CLS formation ability and the “later stage” of endothelial maturation are confirmed by the experiments on HUVECs. HUVECs are mature endothelial cells, demonstrated to posses the ability to rapidly form CLS. However, here we demonstrated that HUVECs are unable to aggregate in 3D spheroids and sprout out, which is an ability that should be attributed to more immature endothelial cells. Nonetheless, HUVEC-containing on-plants stimulated chicken CAM neo-angiogenesis similarly to MPCs. These data are apparently in contrast to the scenario already described, but immunohistochemistry revealed that HLA-positive structures, resembling newly formed capillaries, were detectable exclusively applying MPCs to CAM. This demonstrates the active involvement of MPCs in the morphogenesis of the vessel network. In contrast, HUVEC-containing on-plants produced an increased complexity in the vessel network but no human-derived capillaries were detected, suggesting a paracrine support for neovascularization.

This scenario of “early” and “late” sprouting angiogenesis depicted for MPCs mirrors vasculogenesis. In fact, numerous investigations suggested EPCs to be more heterogeneous than expected and their properties to vary significantly depending on culture methods [25]. In line with MPCs, “early-EPC” CFU-Hill cells lack expression of endothelial marker CD144 while expressing CD45, display low proliferative activity, and are unable to form vessels [7]. Conversely, “late-EPC” ECFCs are CD144^+^ and CD45^neg^, show robust proliferative activity, and form CLS in-vitro [26]. The two distinct populations, cytokine-secreting early EPCs and vessel-forming late EPCs [27], have been proposed to have different roles in neovasculogenesis and vascular repair [28]. More recently, the existence of early EPCs has been questioned. Some authors allocated CFU-Hill cells to the monocytic/macrophagic lineage, due to their failure to form CLS and despite the expression of some endothelial markers and Ac-LDL uptake [29]. Similarly, MPCs and specifically their in-vivo progenitor population, named *Pop#8*, have been hypothesized to belong to the monocytic/macrophagic lineage considering their morphology and phenotype, in particular the expression of CD45 and CD11c. Nonetheless, no genuine hemopoietic colony forming potential has been detected [30]. Here we reported many similarities between early EPCs and MPCs.

We believe that lack of endothelial morphogenesis does not exclude retention of early angiogenic potential. Our results are suggestive of a role for MPCs as primitive progenitor cells and show that their ability to form CLS requires a further differentiation step.

Correlated consecutive stages of differentiation have already been reported in MPC mesengenic induction. MPCs are able to terminally differentiate into adipocytes or osteocytes only through the intermediate precursors P1/P2-MSCs [1, 6]. In the present study we found P2-MSCs to lack angiogenic properties both in-vitro and in-vivo, demonstrating that MPC mesengenic and angiogenic potentials are mutually exclusive. This evidence further supports our hypothesis about the genuine angiogenic potential of bone marrow-derived MPCs. The presence of varying amounts of co-isolated MPCs in heterogeneous adherent bone marrow cultures [1] could be responsible for the controversial data regarding the angiogenic potential of MSC cultures usually ascribed to MSCs [31].

Bone marrow-derived MPCs induced along the mesengenic pathway have been reported to acquire a pericyte-like gene expression pattern characterized by marked expression of *MCAM* (CD146), *ACTA2* (α-SMA), *DESMIN*, *CSPG4* (NG2), and *RGS5* [32]. They could therefore be depicted as a pericytic population characterized by high plasticity [33, 34]. Notably, MPCs showed the potential to initiate new blood vessel formation as well as to contribute to vessel stabilization/maturation, possibly differentiating into pericytes as in the recently proposed mechanistic model of vessel branching [22].

The hypothesis that bone marrow-derived MPCs retain early angiogenic potential is further supported by their constitutive expression of nestin. Much evidence has been reported that nestin expression in vascular endothelial cells is associated with neo-angiogenesis in development, tissue repair, and tumor progression [35]. Nestin has been detected in the endothelial cells of embryonic capillaries [36] as well as in the capillaries of corpus luteum, a tissue highly remodeled by angiogenesis [37]. Proliferating EPCs showed nestin expression as opposed to mature endothelial cells [38]. MPCs revealed a significant nestin downregulation following both mesengenic and angiogenic induction, strongly suggesting that nestin expression can represent a marker for undifferentiated MPCs.

In adult mouse bone marrow, cells expressing GFP in response to a nestin promoter (*Nes*-GFP) and cells expressing *Nes*-cre ER have been shown to exhibit mesenchymal progenitor properties and to constitute an essential hematopoietic stem cell (HSC) niche component [39]. Ono et al. [40] found that vasculature-associated Nes-GFP-expressing cells include not only early osteoprogenitors but also endothelial precursors, supporting the endochondral ossification during bone development. Moreover, *Nes*-cre ER was predominantly expressed in adult bone marrow endothelial cells, including arteriole Cxcl12-producing cells [40].

## Conclusions

Altogether, these data support the idea that both endothelial and nonendothelial nestin-positive cells are important components of the HSC niche and suggest that most cells implicated in defining the HSC niche in bone marrow, including endosteal osteoblasts, endothelial cells, pericytes, and perivascular stromal cells [41–43], could share a common nestin-positive ancestor. According to this scenario, nestin-positive MPCs, due to their ability to differentiate into most cell types sustaining the stromal compartment in-vitro, could play a central role in the establishment and maintenance of the human adult bone marrow microenvironment in-vivo.

## Additional files

Additional file 1: Is a table presenting primer sequences. (DOCX 24 kb)

Additional file 3: Is a figure showing IHC detection of human cells in CAM on-plants. Immunohistochemistry for human HLA-ABC antigen (*brownish stain*) showed human-derived cells within MPC, HUVEC, and P2-MSC on-plants. Applied onto CAM, MPCs showed organized structures mimicking microtubules. Conversely, around HUVEC on-plants, an increase of chicken embryo-derived new vessels was detectable, but human-derived cells appeared not directly involved in microvessel neoformation. P2-MSC on-plants did not show any alteration or remodeling of the CAM tissue, which conserved its three-layer structure. Human cells were embedded within compact and not digested Geltrex™ gel. *Scale bar* = 50 μm. (TIF 3183 kb)

Additional file 4: Is a figure showing representative confocal images of MPCs and HUVECs applied on CAM. Immunofluorescence staining for human HLA-ABC antigen (*green*) revealed human-derived cells involved in tissue remodeling, both with MPC and HUVEC on-plants. Nonetheless, foci of new vessel formation (*arrows*) positive for hCD34 (*red*) were detected only after application of MPC constructs on chicken CAM. Nuclei shown in *blue*, while chicken-derived perfused microvessels were revealed by the presence of nucleated autofluorescent erythrocytes (*pale orange* in the “merge” panels). *Scale bar* = 50 μm. (TIF 3238 kb)

## Abbreviations

Ac-LDL: Acetylated-low density lipoprotein
BM-MNC: Bone marrow-mononuclear cell
BSA: Bovine serum albumin
CAM: Chorioallantoic membrane
CFU: Colony forming unit
CLS: Capillary-like structures
DMEM: Dulbecco’s Modified Minimal Eagle’s Medium
D-PBS: Dulbecco’s phosphate-buffered saline solution
EPC: Endothelial progenitor cell
ECFC: Endothelial colony forming cell
FBS: Fetal bovine serum
GFP: Green fluorescent protein
HLA: Human leukocyte antigens
HUVEC: Human umbilical vein endothelial cell
MPC: Mesangiogenic progenitor cell
MSC: Mesenchymal stromal cell
PhABS: Pooled human AB-type serum
qRT-PCR: Quantitative reverse transcriptase-polymerase chain reaction
SDF-1β: Stromal cell-derived factor 1 beta
SEM: Standard error of the mean
TNF-α: Tumor necrosis factor alpha
VEGF: Vascular endothelial growth factor
