# Supplementary material for: Human adult mesangiogenic progenitor cells reveal an early angiogenic potential, which is lost after mesengenic differentiation
Source: Stem Cell Res Ther. 2017 May 2;8:106. doi: 10.1186/s13287-017-0562-x (PMC5414340; doi:10.1186/s13287-017-0562-x)
Supplement: Supplementary file 3 — Is a table presenting primer sequences. (DOCX 24 kb) [file 13287_2017_562_MOESM1_ESM.docx]

| **NCBI Ref#** | **Gene Name** | **Primer Pair** | | **Amplicon** |
| --- | --- | --- | --- | --- |
|  |  |  |  | **length** |
|  |  |  |  |  |
| NM_000442 | PECAM-1 | *sense* | GAACCTGTCCTGCTCCATC | 231 bp |
|  |  | *anti-sense* | TCAAACTGGGCATCATAAGAAAT |  |
|  |  |  |  |  |
| NM_000214 | JAG1 | *sense* | CAATGACTTCTACTGTGACT | 179 bp |
|  |  | *anti-sense* | TGTTTCGGGCTATGTTAC |  |
|  |  |  |  |  |
| NM_006617 | NESTIN | *sense* | GTTGGAACAGAGGTTGGAG | 250 bp |
|  |  | *anti-sense* | GAGGGAAGTCTTGGAGCC |  |
|  |  |  |  |  |
| NM_001040060 | SPP1 | *sense* | GCCGAGGTGATAGTGTGGTT | 101 bp |
|  |  | *anti-sense* | TGAGGTGATGTCCTCGTCTG |  |
|  |  |  |  |  |
| NM_001253357 | TIE1 | *sense* | CCAAAGACAGGATACAGTG | 110 bp |
|  |  | *anti-sense* | AACAAAGGCTCAGGACAG |  |
|  |  |  |  |  |
| NM_020404 | TEM1 | *sense* | CGAGTGTTATTGTAGCGA | 137 bp |
|  |  | *anti-sense* | CTTCATCTTCCTCATCCT |  |
|  |  |  |  |  |
| NM_000459 | TEK | *sense* | GCTTGGACCCTTAGTGACATTCTT | 199 bp |
|  |  | *anti-sense* | ACTGAGTGATGGTGGCATTCTT |  |
|  |  |  |  |  |
| NM_019074 | DLL4 | *sense* | TGCGGTTACACAGTGAAAAG | 125 bp |
|  |  | *anti-sense* | TACCTCCGTGGCAATGAC |  |
|  |  |  |  |  |
| NM_002253 | KDR | *sense* | CCGCAGAGTGAGGAAGGAG | 190 bp |
|  |  | *anti-sense* | CCGTAGGATGATGACAAGAAGTAG |  |
|  |  |  |  |  |
| NM_000552 | VWF | *sense* | CCAGCTTCTGAAGAGCACCTC | 240 bp |
|  |  | *anti-sense* | ACACACTGCCTATACTCCATACCA |  |
|  |  |  |  |  |
| NM_001613 | ACTA2 | *sense* | GTTACTACTGCTGAGCGTGAG | 109 bp |
|  |  | *anti-sense* | CAAGGGAGGATGAGGATGC |  |
|  |  |  |  |  |
| NM_001927 | DES | *sense* | TCAACTTCCGAGAAACCA | 143 bp |
|  |  | *anti-sense* | CTGTCTTTAGAGCACTTCAT |  |
|  |  |  |  |  |
| NM_001195303 | RGS5 | *sense* | CTTGAGTTCTGGATTGCCTGTGA | 147 bp |
|  |  | *anti-sense* | TGTGATGTCCTTAGTGAAGTGGTC |  |
|  |  |  |  |  |
| NM_006500 | MCAM | *sense* | AGAGACAACCAACGACAA | 145 bp |
|  |  | *anti-sense* | AGACACATAGTTCACCAGTA |  |
|  |  |  |  |  |
| NM_002701 | POU5F1 | *sense* | CCGTGAAGCTGGAGAAGGAGAAG | 198 bp |
|  |  | *anti-sense* | AGCGGCAGATGGTCGTTTGG |  |
|  |  |  |  |  |
| NM_001897 | CSPG4 | *sense* | TGGAGAATGGTGGAAGAG | 137 bp |
|  |  | *anti-sense* | AGGACAGTGACAGTGAAG |  |
|  |  |  |  |  |
| NM_001198688 | LEPR | *sense* | TTATATGTTCTGCCTGAAGTGTT | 146 bp |
|  |  | *anti-sense* | AGGAGAGTGTCGTTGAGTT |  |
|  |  |  |  |  |
| NM_001795 | CDH5 | *sense* | AGCCCAAAGTGTGTGAGAA | 151 bp |
|  |  | *anti-sense* | GCCGTGTTATCGTGATTATCC |  |
|  |  |  |  |  |
| NM_001270951 | RANK | *sense* | CTGGGACGGTGCTGTAAC | 164 bp |
|  |  | *anti-sense* | GCCTTGCCTGTATCACAAAC |  |
|  |  |  |  |  |
| NM_003701 | RANKL | *sense* | AGCAGAGAAAGCGATGGT | 132 bp |
|  |  | *anti-sense* | GACAGACTCACTTTATGGGAAC |  |
|  |  |  |  |  |
| NM_033023 | PDGFA | *sense* | GAAGAGAAGCATCGAGGAAG | 105 bp |
|  |  | *anti-sense* | ATCAGGAAGTTGGCGGAC |  |
|  |  |  |  |  |
| NM_033016 | PDGFB | *sense* | AGTGTGTGGGCAGGGTTA | 112 bp |
|  |  | *anti-sense* | CCGTCCGAATCAGGCATC |  |
|  |  |  |  |  |
| NM_002184 | GP130 | *sense* | AATGGCAGCATACACAGATGAAGG | 149 bp |
|  |  | *anti-sense* | AGCAGAACAGCACTCCCAGAAG |  |
|  |  |  |  |  |
| NM_002310 | LIFR | *sense* | GGGCTCATCACCACCTTCC | 164bp |
|  |  | *anti-sense* | CCGACCGAGACGAGTTACAC |  |
|  |  |  |  |  |
| NM_003994 | SCF | *sense* | CGCTGCCTTTCCTTATGA | 232 bp |
|  |  | *anti-sense* | ATCTCGCTTATCCAACAATGA |  |
|  |  |  |  |  |
| NM_004348 | RUNX2 | *sense* | CCAACCCACGAATGCACTATC | 91 bp |
|  |  | *anti-sense* | TAGTGAGTGGTGGCGGACATAC |  |
|  |  |  |  |  |
| NM_001118888 | ANGPT2 | *sense* | CAAATCAGGACACACCAC | 178 bp |
|  |  | *anti-sense* | ACCAAATCCCACTTTATATTCTT |  |
|  |  |  |  |  |
| NM_002020 | FLT4 | *sense* | CACATCACAGGCAACGAG | 139bp |
|  |  | *anti-sense* | GGTAGTCCCAGTCAAAGG |  |
|  |  |  |  |  |
| NM_006474 | PDPN | *sense* | ACAGTGTAACAGGCATTC | 163bp |
|  |  | *anti-sense* | GACAAACCATCTTTCTCAAC |  |
|  |  |  |  |  |
| NM_002763 | PROX1 | *sense* | AATGACTTTGAGGTTCCAGAGAG | 175bp |
|  |  | *anti-sense* | GGCAGTTCGGGGATTTGA |  |
|  |  |  |  |  |
| NM_012423 | RPL13A | *sense* | CCTGGAGGAGAAGAGGAAAGAGA | 126 bp |
|  |  | *anti-sense* | TTGAGGACCTCTGTGTATTTGTCAA |  |
|  |  |  |  |  |
| NM_001101 | ACTB | *sense* | CGCCGCCAGCTCACCATG | 101 bp |
|  |  | *anti-sense* | CACGATGGAGGGGAAGACGG |  |
