# Supplementary figures and images for: Human adult mesangiogenic progenitor cells reveal an early angiogenic potential, which is lost after mesengenic differentiation
Source: Stem Cell Res Ther. 2017 May 2;8:106. doi: 10.1186/s13287-017-0562-x (PMC5414340; doi:10.1186/s13287-017-0562-x)

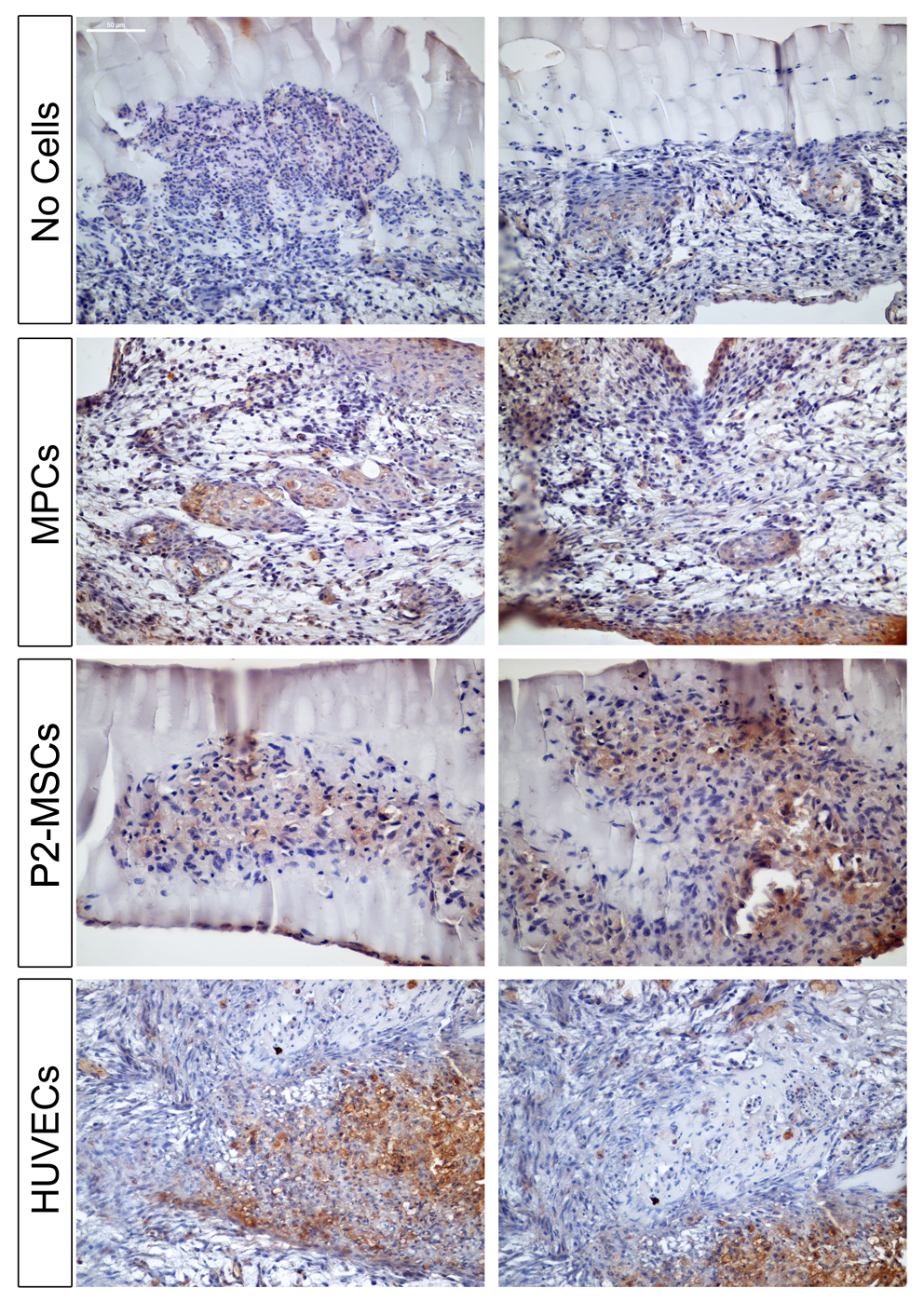

Supplement: Supplementary file 4 — Is a figure showing IHC detection of human cells in CAM on-plants. Immunohistochemistry for human HLA-ABC antigen (brownish stain) showed human-derived cells within MPC, HUVEC, and P2-MSC on-plants. Applied onto CAM, MPCs showed organized structures mimicking microtubules. Conversely, around HUVEC on-plants, an increase of chicken embryo-derived new vessels was detectable, but human-derived cells appeared not directly involved in microvessel neoformation. P2-MSC on-plants did not show any alteration or remodeling of the CAM tissue, which conserved its three-layer structure. Human cells were embedded within compact and not digested Geltrex™ gel. Scale bar = 50 μm. (TIF 3183 kb) [file 13287_2017_562_MOESM3_ESM.tif]

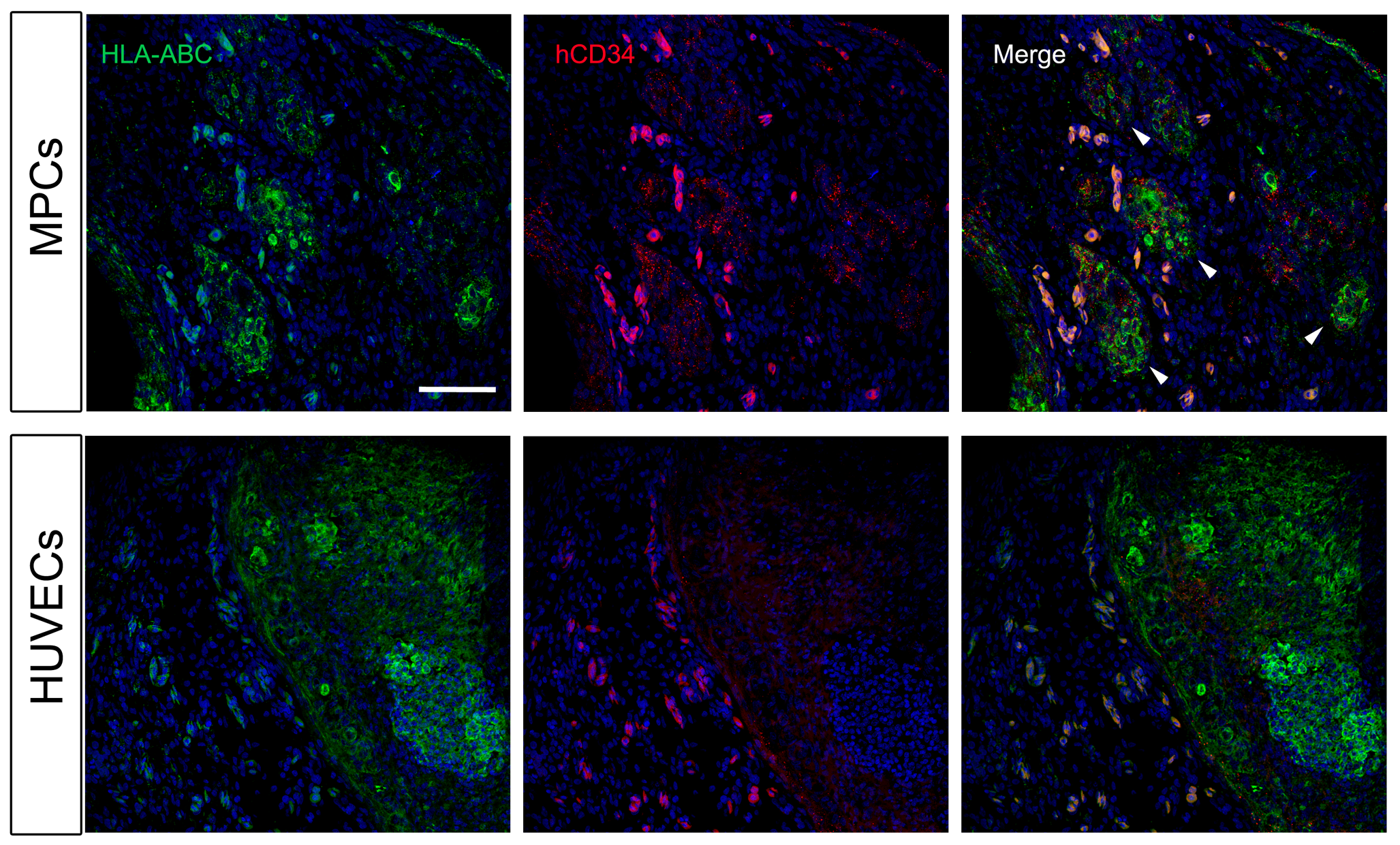

Supplement: Supplementary file 5 — Is a figure showing representative confocal images of MPCs and HUVECs applied on CAM. Immunofluorescence staining for human HLA-ABC antigen (green) revealed human-derived cells involved in tissue remodeling, both with MPC and HUVEC on-plants. Nonetheless, foci of new vessel formation (arrows) positive for hCD34 (red) were detected only after application of MPC constructs on chicken CAM. Nuclei shown in blue, while chicken-derived perfused microvessels were revealed by the presence of nucleated autofluorescent erythrocytes (pale orange in the “merge” panels). Scale bar = 50 μm. (TIF 3238 kb) [file 13287_2017_562_MOESM4_ESM.tif]
